# CpG Methylation of Protein Prenyltransferase Genes *FNTA*, *FNTB*, *PGGT1B* and *RABGGTA* in Cancer Cell Lines †

**DOI:** 10.3390/epigenomes10010017

**Published:** 2026-03-04

**Authors:** Dominik Jung, Daniel Diehl, Anna Hagemann, Hagen Sjard Bachmann

**Affiliations:** Institute of Pharmacology and Toxicology, Center for Biomedical Education and Research (ZBAF), School of Medicine, Faculty of Health, Witten/Herdecke University, 58453 Witten, Germany; dominik.jung@uni-wh.de (D.J.); daniel.diehl@uni-wh.de (D.D.); anna.hagemann@uni-wh.de (A.H.)

**Keywords:** protein prenylation, prenyltransferases, DNA methylation, mRNA expression, cancer, pyrosequencing, qPCR, regulation

## Abstract

**Background:** Protein prenylation is crucial for the function of hundreds of proteins. Aberrant protein prenylation can be caused by the aberrant expression of prenyltransferases (PTases), which has been reported for multiple cancer entities. The reasons for aberrant PTase expression in cancer have not yet been investigated. **Methods:** We analyzed CpG methylation within promoter-associated CpG islands of the PTase genes *FNTA*, *FNTB*, *PGGT1B*, and *RABGGTA* via bisulfite conversion and pyrosequencing to assess its role in PTase expression and gain deeper insight into the regulation of protein prenylation in cancer. We used DNA from three benign controls (whole blood samples, peripheral blood mononuclear cells, and HEK293) and 19 human cancer cell lines from various origins to assess DNA methylation within PTase gene promoter-associated CpG islands. For a subset of these cell lines, we measured mRNA expression via qPCR and correlated it with DNA methylation. **Results:** Methylation across all PTase genes ranged from 1.9 ± 0.9% to 11.4 ± 4.0% (mean methylation ± standard deviation) in benign cells, and 2.3 ± 1.0% to 16.0 ± 5.4% in cancer cells. DNA methylation and mRNA expression of *PGGT1B* correlated inversely (PCC = −0.75; *p* = 0.005). **Conclusions:** We saw no general differences between benign and malignant cells, but observed significant differences between non-malignant controls and multiple individual cancer cell lines regarding the methylation of PTase genes. This was prominently seen in *PGGT1B* in Caki-1 cells, raising the possibility that DNA methylation is involved in the dysregulation of PTase expression in cancer.

## 1. Introduction

Protein prenyltransferases (PTases) are a group of conserved heterodimeric enzymes comprising farnesyltransferase (FTase) and geranylgeranyltransferase type I and II (GGTase I and GGTase II) [1]. They use the mevalonate pathway intermediates farnesyldiphosphate (FPP) or geranylgeranyldiphosphate (GGPP) for the posttranslational modification of hundreds of proteins (among them many encoded by proto-oncogenes, e.g., Ras), which is crucial for the proper function and localization of those proteins. A third geranylgeranyltransferase (GGTase III) has been identified, but only a few of its targets are currently known [2,3]. The aberrant expression of FTase, GGTase I, and GGTase II has frequently been observed in cancer, including ovarian carcinoma [4,5], skin carcinoma [5,6], glioma [7], and liver cancer [8] (Table 1). However, the causes of divergent PTase expression in cancer remain mostly unknown.

**Figure 1:**
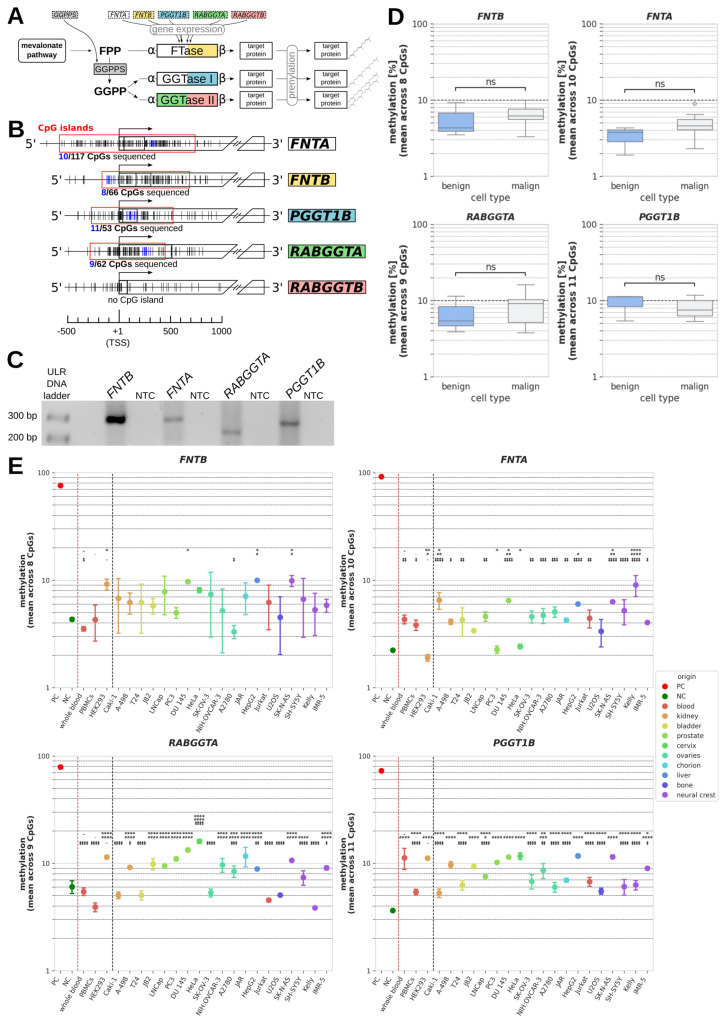
**DNA methylation of PTase genes.** (**A**) Schematic summary of protein prenylation pathways. (**B**) Relative positions of CpGs surrounding the transcription start sites (TSSs) of prenyltransferase (PTase) genes. Individual CpGs are displayed as vertical lines with CpGs sequenced in this study being highlighted in blue. Regions defined as CpG islands are indicated by a red frame, and the total number of CpGs and the number of sequenced CpGs within each CpG island are shown below. (**C**) Representative DNA bands of analyzed PCR products yielded after the bisulfite conversion of genomic DNA samples and subsequent bisulfite specific PCRs (BSPs) of regions containing the sequenced CpGs highlighted in Figure 1B. ULR: ultra-low range; NTC: no template control. (**D**) Boxplot representation of mean methylation across all sequenced CpGs within PTase genes *FNTB*, *FNTA*, *RABGGTA*. and *PGGT1B* in benign cells (whole blood, PBMCs, and HEK293) vs. cancer cell lines. (**E**) Mean methylation of individual benign and malignant cell samples summarized in Figure 1D. Negative (NCs) and positive controls (PCs) were demethylated and methylated, respectively, prior to BSP. *p* values of significant difference between a sample and different reference cells are indicated by different symbols for whole blood (*), PMBCs (^#^), and HEK293 (^‡^); */^#^/^‡^: *p* < 0.05; **/^##^/^‡‡^: *p* < 0.01; ***/^###^/^‡‡‡^: *p* < 0.001; ****/^####^/^‡‡‡‡^: *p* < 0.0001; ns (not significant): *p* ≥ 0.05.

PTases catalyze the transfer of either farnesyl or geranylgeranyl residues to their target proteins, which alter the targets’ lipophilicity and can act as membrane anchors or recognition motifs for protein–protein interactions (Figure 1A). This process, called protein prenylation, is dependent on the availability of the substrates FPP and GGPP, and the concentration of PTases. Another enzyme, GGPP synthase (GGPPS), is responsible for the conversion of FPP to GGPP. Each PTase consists of one α- and one β-subunit (each coded by its own gene), with the gene product of *FNTA* serving as the α-subunit of both FTase and GGTase I. Further PTase genes are *FNTB* (β-subunit of FTase), *PGGT1B* (β-subunit of GGTase I), *RABGGTA*, and *RABGGTB* (α- and β-subunit of GGTase II, respectively). Each PTase has its own spectrum of target proteins, but as their targets partially overlap, an altered ratio of FPP to GGPP can decide whether certain proteins are farnesylated or geranylgeranylated, thus modifying their biophysical characteristics [10]. PTase gene expression represents the other main factor deciding to what degree a target protein population is prenylated. Importantly, a distorted proportion of prenylation results in the aberrant activity of the target protein population, which has been linked to the development and progression of diseases. For instance, there are cases where a pathologically high degree of prenylation leads to the overactivity of certain protein targets and, consequently, to overactive signaling pathways that contribute to the emergence of cancer, while in other cases, inhibited protein prenylation can result in mislocalized protein targets associated with inflammatory processes [9,11].

DNA methylation of CpG dinucleotides represents one major epigenetic mechanism regulating gene expression that is frequently altered in cancer, especially within CpG islands covering regulatory regions [12]. For GGPPS, a connection between differentially methylated promoters, altered gene expression, altered protein prenylation, and disease progression has been demonstrated [13]. DNA methylation of the *GGPPS* promoter following juvenile mumps infection results in an imbalance of both prenyl substrates, aberrant protein prenylation, and consequently adult infertility [13]. Furthermore, in plant physiology, *GGPPS* expression is altered due to DNA methylation during the root growth stages of red sage (*Salvia miltiorrhiza*), indicating a regulatory mechanism conserved far beyond the mammalian context [14].

Considering that differences in DNA methylation are often contributing to aberrant gene expression in cancer, and that DNA methylation is involved in the regulation of *GGPPS* expression under both pathological and physiological conditions, we hypothesized that human PTase promoters might also contain CpG islands that exhibit distinct methylation patterns in cancer cells, thus altering PTase expression in cancer. We therefore looked for CpG islands in the gene promoters of FTase (*FNTA* and *FNTB*), GGTase I (*FNTA* and *PGGT1B*) and GGTase II (*RABGGTA* and *RABGGTB*). Subsequently, we assessed DNA methylation within the identified CpG islands via bisulfite pyrosequencing in 19 cancer cell lines (including neuroblastoma, ovarian, and liver cancer) and compared it to the methylation in whole blood samples, peripheral blood mononuclear cells (PBMCs), and HEK293 cells. Finally, we assessed the mRNA expression of these genes in a subset of these cell lines via qPCR and absolute quantification, and checked for correlations between DNA methylation and mRNA expression.

## 2. Results

### 2.1. Characterization of CpG Islands

CpG islands were found to cover the transcription start sites (TSSs) of *FNTA*, *FNTB*, *PGGT1B*, and *RABGGTA* (but not of *RABGGTB*) containing 117 CpGs, 66 CpGs, 53 CpGs, and 62 CpGs, respectively (Figure 1B). We amplified a ROI within each of these CpG islands, yielding an amplicon of a specific size and number of covered CpG dinucleotides for each gene, specifically: *FNTB*: 284 bp, 8 CpGs covered; *FNTA*: 290 bp, 10 CpGs covered; *RABGGTA*: 221 bp, 9 CpGs covered; *PGGT1B*: 273 bp, 11 CpGs covered. Each PCR product showed one specific band of the expected size on agarose gel, as is required for pyrosequencing (Figure 1C).

### 2.2. Analysis of DNA Methylation

The mean methylation of the sample cells ranged from 1.9 ± 0.9% to 16.0 ± 5.4% over all analyzed PTase genes and cell lines, and standard deviation was between 0.8 and 7.1 (Table 2). Mean methylation in the minimally methylated negative controls ranged from 2.2 ± 1.1% to 6.0 ± 2.4%, whereas in maximally methylated positive controls, mean methylation ranged from 72.7 ± 14.1% in *PGGT1B* to 91.1 ± 7.7% in *FNTA*, showing that none of the four PTase genes could be regarded as maximally methylated in any of the analyzed sample cells.

Summarized comparisons of benign (whole blood, PBMCs, and HEK293) and malign samples (all cancer cell lines) revealed no significant differences in DNA methylation between the two groups (Figure 1D). Nonetheless, to detect potential differences between individual cancer cell lines and non-malignant cells, we additionally compared the methylation in each cancer cell line to each benign control in an ANOVA (Figure 1E and Table 2).

For *FNTB*, there was no cell line for which a significant difference could be detected to each of the three benign controls at once. For *FNTA*, *RABGGTA*, and *PGGT1B*, there were several cell lines showing significant differences in DNA methylation (in percentage points, pp) compared to whole blood samples, PBMCs, and HEK293 cells (Table 2, bold highlighting). The methylation of *FNTA* was significantly higher in Caki-1, DU 145, SK-N-AS, and Kelly cells than in each control, with the strongest differences being observed for Kelly cells (whole blood: *p* < 0.0001; 4.7 pp (95%-CI: 2.8;6.6); PBMCs: *p* < 0.0001; 5.2 pp (95%-CI: 3.3;7.1); HEK293: *p* < 0.0001; 7.1 pp (95%-CI: 5.3;9.0); Table 2). In contrast, most cell lines with a significant difference to every benign control regarding the methylation of *RABGGTA* and *PGGT1B* showed higher methylation than one or two benign controls, but lower methylation than the other control(s), due to the relatively wide interval spanned by the controls. Additionally, there were several cancer cell lines for each gene in which methylation differed from only one or two non-malignant controls. In total, the absolute values of the significant differences in methylation between benign and malign cells ranged from 1.9 to 12.1 pp mean difference and contained both negative values (less methylation than benign cells) and positive values (higher methylation than benign cells; see Table 2).

**Figure 2:**
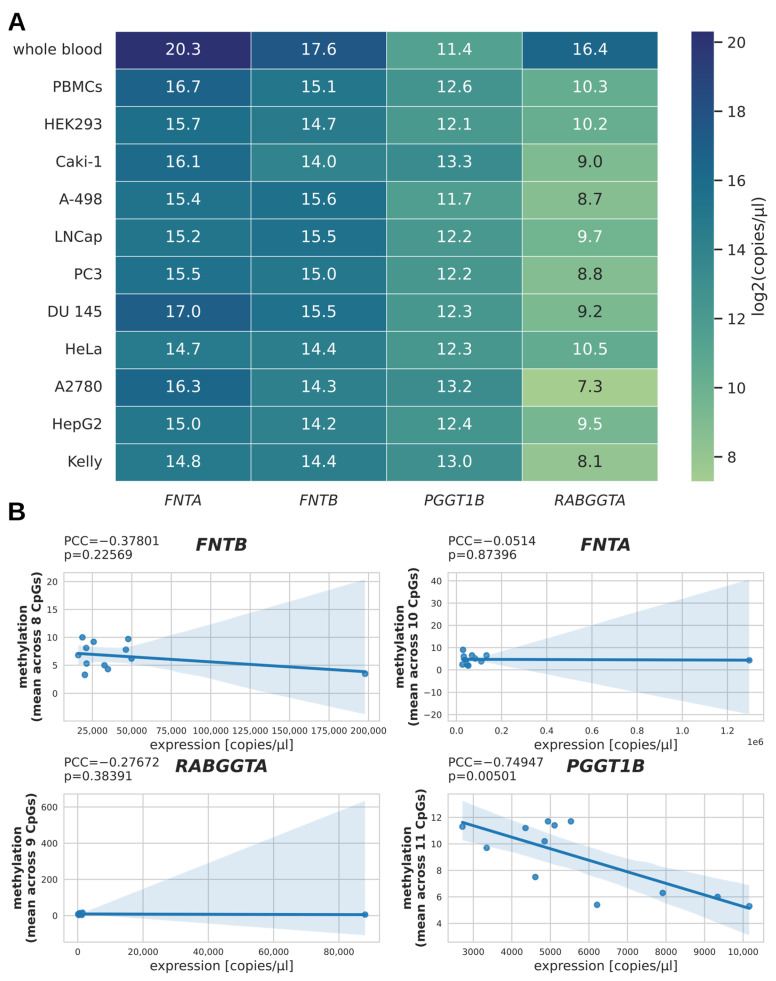
**mRNA expression of PTase genes.** (**A**) Heatmap showing the transcript numbers of PTase genes for selected cell lines as quantified by qPCR. For better representation of these values in a heatmap, the binary logarithm of the transcript number per µL was taken before mapping. (**B**) Scatterplots for each gene plotting DNA methylation against the number of transcripts per µL in order to visualize potential correlation between DNA methylation and gene expression.

To evaluate the relation between promoter methylation and gene expression, we estimated the number of transcripts per µL for the benign controls and selected cancer cell lines via qPCR measurement and absolute quantification, and represented the binary logarithm of these values in a heatmap (Figure 2A). The subset of samples for qPCR analysis included ten cancer cell lines, PBMCs, and whole blood, resulting in a total of twelve pairs of RNA/DNA samples (*n* = 12). Interestingly, the expression of *FNTA*, *FNTB*, and *RABGGTA* (but not of *PGGT1B*) in whole blood samples was about an order of magnitude higher than in the second highest expressing samples. To test whether DNA methylation and expression correlated for the four PTase genes, we plotted the mean methylation against the number of transcripts per µL and calculated the Pearson correlation coefficient (PCC; Figure 2B). As the PCC can be sensitive to outliers [15], we compared the PCC calculated from all data with the PCC when excluding the whole blood samples as outliers. This revealed an inverse correlation between mean methylation and number of transcripts for *PGGT1B* only, both when regarding all samples (PCC = −0.75; *p* = 0.005; *n* = 12; Figure 2B) and when excluding the whole blood sample as an outlier (PCC = −0.73; *p* = 0.011; *n* = 11; cf. Supplementary Figure S1).

## 3. Discussion

In this pilot study, we investigated the hypothesis that the differential expression of PTases in cancer is influenced by the differential DNA methylation of PTase genes. Pyrosequencing allowed us to resolve the methylation state of selected regions within the PTase promoters in great detail, even though this targeted approach lacks the amount of data generation reached by hypothesis-free omics approaches.

None of the cell lines analyzed in this study were predominantly methylated at the CpG islands overlapping with the promoters of *FNTB*, *FNTA*, *RABGGTA*, and *PGGT1B,* but instead exhibited methylation values clearly more akin to the negative controls than to the positive controls. When comparing the DNA methylation of the group of benign samples (whole blood, PBMCs, and HEK293) in these regions to the group of cancer cell lines, no significant difference could be detected. However, comparisons between individual cancer cell lines and each of the three non-malignant reference cells revealed slight, yet statistically significant differences regarding the methylation of each PTase gene for many cancer cell lines. For *FNTA*, *RABGGTA*, and *PGGT1B*, there even were several cancer cell lines that exhibited DNA methylation that was significantly different from each of the three benign controls. In all these cases, the methylation of *FNTA* was uniformly higher in cancer cells than in the benign controls, the differences being most pronounced in renal clear cell carcinoma cell line Caki-1 and neuroblastoma cell line Kelly. In contrast, the methylation of *RABGGTA* and *PGGT1B* in cells with significant differences to all three controls was between that of the most and the least methylated control. This illustrates the difficulties in comparing methylation in cancer cell lines to controls of non-matched origin as different controls sometimes differed significantly from each other, thus hampering the identification of the best representative of a non-malignant reference condition. For instance, the differences observed between the HEK293 and Kelly cells may represent tissue-specific rather than cancer-specific methylation patterns. Nonetheless, we refrained from increasing the number of benign cell lines or primary tissue controls due to the generally low DNA methylation of PTase promoters observed among all cancer cell lines. Consequently, our main focus was the comparisons between HEK293 and the kidney cancer cell lines Caki-1 and A-498, despite significant differences between many other individual cancer cell lines and controls. It should be noted, however, that even the differences between these renal cell lines might be influenced by their different developmental states.

Regarding PTase expression, we showed that only in the case of *PGGT1B* did DNA methylation correlate with mRNA expression. Recent reviews indicate that promoter DNA methylation represents only one regulatory layer within a broader chromatin context. Its relationship with gene expression is therefore context-dependent and not necessarily linear [16,17,18]. The lack of correlation for *FNTA*, *FNTB*, and *RABGGTA* may thus reflect additional regulatory mechanisms beyond promoter methylation alone. One possible explanation, for instance, could be distinct levels of relevant transcription factors, which can mask the effect of DNA methylation on mRNA expression as long as the gene’s expression is not completely silenced. However, studies on regulatory mechanisms in the context of PTases are scarce. To our knowledge, single-nucleotide polymorphisms of *FNTB* are currently the only known genetic modifications of PTase genes contributing to the activity of the corresponding PTase [19]. Thus far, epigenetic modifications of PTase genes, like histone modifications, have not specifically been investigated. There is, however, evidence suggesting a role of non-coding RNA in the regulation of PTase activity [20,21].

Of note, the DNA methylation of *PGGT1B* was higher in HEK293 cells than in Caki-1 cells, while its mRNA expression was lower. This is in line with public expression data for HEK293 and Caki-1 (https://www.proteinatlas.org/ENSG00000164219-PGGT1B/cell+line, accessed on 24 July 2025) and may indicate an epigenetic regulatory mechanism relevant for *PGGT1B* expression in renal cell carcinoma. This may be of particular interest as *PGGT1B* expression is a validated prognostic factor in certain kidney tumors (https://www.proteinatlas.org/ENSG00000164219-PGGT1B/cancer/renal+cancer, accessed on 24 July 2025).

For the interpretation of these results, a couple of limitations of this pilot study have to be considered. These limitations arise from the screening approach of the study setting and the method employed and necessitate the validation of the results by complementary methods in subsequent studies.

First, the influence of cell culturing and cell line establishment on DNA methylation is not entirely clear. It has been known for a long time that DNA methylation in cell lines can differ from DNA methylation in original tissue [22]. Such alterations may arise from the use of cell culture media or changes in the microenvironment [23]. However, other studies comparing DNA methylation in primary tumors and corresponding cancer cell lines have indicated that, in the majority of cases, aberrant DNA methylation in cancer cell lines is also present in the primary tumors [24,25]. Thus, studying cancer-specific DNA methylation in cell lines appears generally viable, although the results from such studies should be validated in primary tumor tissue to exclude any artifacts potentially arising from cell culture. In the present study, we used HEK293 as the only non-malignant cell line. HEK293 is an embryonic, adenovirus-transformed cell line of renal origin and may thus not accurately represent the physiological methylation state of adult tissue. The whole blood and PBMC samples (unlike all other cell samples) represented a heterogeneous mix of different cell populations present in the blood stream that was not clearly defined. On the one hand, this limits the reproducibility of the measured methylation values; on the other hand, this may actually increase their validity as a non-malignant control as these samples represent a broader spectrum of healthy cells and are not limited to one specific cell type. However, in a more focused cell panel, matched non-malignant controls from the same tissue as each investigated cancer cell line would certainly increase the power of future studies.

Second, the method used in this study, pyrosequencing, allowed the analysis of a relatively small portion of each CpG island. Even though the methylation status of few or even single CpGs within a CpG island is mostly representative for the methylation status of the whole CpG island [26], there can be relevant exceptions from this principle [27].

Furthermore, a recent study reported the differential methylation of a CpG located in intron 2 of PTase gene *RABGGTB* (cg08702915) in individuals with autism spectrum disorder [28]. For the sake of comparability, we focused on the analysis of CpGs within promoter-associated CpG islands and therefore excluded *RABGGTB* from this study. It should be noted, however, that aberrant DNA methylation that affects gene expression is not necessarily confined to CpG islands or gene promoters, even though this kind of methylation represents one of the most noted and most studied types of DNA methylation [29]. Therefore, using long-read sequencing techniques to investigate larger PTase gene portions, and including all PTase genes may yield more informative results and would have the additional advantage of avoiding the need for DNA preparation and, thus, the risk of PCR bias.

Lastly, we investigated the direct association of DNA methylation with gene expression in this study. Even though higher expression of PTases, in turn, generally correlates with higher protein prenylation, future studies are needed to validate a functional relationship between DNA methylation and PTase expression on the one hand, and protein prenylation and disease progression in cancer on the other hand. Ideally, such investigations would include the identification of the specific PTase targets causal for differences between benign and malignant cells, as most PTases can target dozens of different proteins. Despite these limitations, the current study indicates the existence of differential DNA methylation in specific cancer cell lines affecting PTase expression, and delivers important clues for the selection of PTase genes and cell lines for investigation via more elaborate yet more costly and time-consuming techniques.

## 4. Materials and Methods

### 4.1. Identification of CpG Islands and Primer Design

We examined the human PTase genes *FNTA*, *FNTB*, *PGGT1B*, *RABGGTA*, and *RABGGTB* regarding the presence of CpG islands (as defined by Takai and Jones [30]) using the Genome Data Viewer from the National Center for Biotechnology Information (assembly GRCh38.p14; https://www.ncbi.nlm.nih.gov/genome/gdv/?org=homo-sapiens, accessed on 8 November 2023). We then designed multiple pairs of oligonucleotides for the amplification of regions within the identified CpG islands via polymerase chain reaction (PCR) after bisulfite conversion using the PyroMark Assay Design software (version 2.0.1.15; Qiagen, Hilden, Germany). We selected one oligonucleotide pair per gene based on PCR product specificity (as evaluated by agarose gel electrophoresis), as well as signal strength and reproducibility during pyrosequencing.

### 4.2. Cell Culture

HEK293, HeLa, PC3, and HepG2 cells were cultured in Dulbecco’s Modified Eagle Medium (DMEM; PAN-Biotech, Aidenbach, Germany), with HepG2 cells being supplemented with 1% non-essential amino acids and 2 mmol/L glutamine. A-498, DU-145, and J82 cells were cultured in Eagle’s Minimum Essential Medium (EMEM; PAN-Biotech, Aidenbach, Germany). A2780, Kelly, Jurkat, JAR, NIH:OVCAR-3, LNCaP, SK-N-AS, IMR-5, and SH-SY5Y cells were cultured in Roswell Park Memorial Institute (RPMI) 1640 medium (PAN-Biotech, Aidenbach, Germany). U2OS, Caki-1, T24, and SK-OV-3 cells were cultured in McCoy’s 5A Medium (PAN-Biotech, Aidenbach, Germany), with T24 and SK-OV-3 cells being supplemented with 2 mmol/L glutamine. Each medium was additionally supplemented with 10% fetal bovine serum (FBS), 100 U/mL penicillin, and 100 µg/mL streptomycin, and the cells were kept in a humidified incubator at 37 °C and 5% CO_2_. All cell lines were cultured in T-75 cell culture flasks (Sarstedt, Nümbrecht, Germany) using 10 mL of cell culture medium per flask, resulting in approximately 5–10 × 10^6^ cells per flask at the time of cell harvest.

### 4.3. DNA Extraction, Processing of Controls, and Bisulfite Treatment

For DNA extraction, cells were grown to ~90% confluence, detached with trypsin/EDTA (PAN-Biotech¸ Aidenbach, Germany), and pelletized via centrifugation at 300 x g for 5 min. DNA was then extracted from cell pellets (or from a whole blood sample) using the DNeasy Blood & Tissue Kit (Qiagen, Hilden, Germany), and concentration and purity were determined via the ratio of absorbance at 260 nm and 280 nm as measured on an Infinite M Plex plate reader in combination with a NanoQuant plate (Tecan Infinite200 Pro, Tecan Group AG, Männedorf, Switzerland). The whole blood and PBMC samples were acquired commercially from Research Donors Ltd. (London, UK) as anonymized samples including the healthy donors’ consent for using the samples in biomedical research. We used maximally and minimally methylated, bisulfite-converted DNA from the EpiTect PCR Control DNA Set (Qiagen, Hilden, Germany) as positive controls (PCs) and negative controls (NCs), respectively. All non-control DNA samples were bisulfite-treated according to the manufacturer’s instructions using the EpiTect Bisulfite Kit (Qiagen, Hilden, Germany).

### 4.4. Bisulfite Sequencing PCR and Pyrosequencing

Following bisulfite conversion, the regions of interest (ROIs) within the promoters of *FNTA*, *FNTB*, *PGGT1B*, and *RABGGTA* were amplified via bisulfite sequencing PCR (BSP) using Taq DNA Polymerase (Master Mix RED, AMPLIQON, Odense, Denmark). DNA samples or controls served as templates, and the following oligonucleotides were used as primers: *FNTA_forward*: 5′-GTTTTTTTATGTTTTGTATAGGTAA-3′; *FNTA_reverse*: 5′-biotin-ACAATCAAAAATACAACAATTTCC-3′; *FNTB_forward*: 5′-TATTGGGTAGAGGAGTTGTTG-3′; *FNTB_reverse*: 5′-biotin- ATACCCCAAAATACACAATAATTCTTTTAA-3′; *PGGT1B_forward*: 5′-biotin-TTATTGAGGATGAGAGGTTAGTAGG-3′; *PGGT1B_reverse*: 5′-AAACCCACTTAAAACCAATCAATAC-3′; *RABGGTA_forward*: 5′-biotin-TTTGGGGAGTAGAGTGGTT-3′; *RABGGTA_reverse*: 5′-CCCTACTAACTCCCCCCACAACTATATCC-3′. The primer stock solutions had a concentration of 10 µmol/L. The total volume of each PCR was 30 µL, containing 15 µL Taq Mix, 0.5 µL DNA template, 1 µL forward and reverse primer each (*RABGGTA*: 0.8 µL each), 1 µL DMSO (except for *FNTB*), and 11.5–12.5 µL H_2_O. Each PCR was started with an initial denaturation at 95 °C for 3 min, followed by 45 cycles of 30 s at 95 °C, 30 s at a ROI-specific annealing temperature (*FNTA*: 49.1 °C; *FNTB*: 58.0 °C; *PGGT1B*: 54.1 °C; *RABGGTA*: 57.2 °C), and 20 s elongation at 72 °C, and concluded with 5 min at 72 °C. For each run, at least one no-template control (NTC) was included, lacking a DNA template. After BSP, the PCR products were cleaned up using the ROTI Prep PCR Purification Kit (Carl Roth, Karlsruhe, Germany). Subsequently, part of the PCR products was visualized on a 2.5% agarose gel using the Ultra Low Range (ULR) DNA Ladder (Thermo Fisher Scientific, Waltham, MA, USA) for size comparison. The remaining PCR products were immobilized on streptavidin-coated sepharose beads, treated with 0.2 mol/L NaOH for strand separation and annealed to the respective sequencing primer (*FNTA_seq*: 5′-GAGGTTTAGAGGTTTTAAGA-3′; *FNTB_seq*: 5′- AGGAGTTGTTGTAAGAG-3′; *PGGT1B_seq*: 5′-TACCAACCTAACTAACTATA-3′; *RABGGTA_seq*: 5′-AAACTTTACACCTTCCACAAAAAA-3′) for 2 min at 80 °C before sequencing on a PyroMark Q24 device (Qiagen, Hilden, Germany). The sequencing of all four ROIs was performed in at least three replicates for each cell line. All oligonucleotides were purchased from Eurofins Genomics, Ebersberg, Germany.

### 4.5. RNA Extraction and cDNA Synthesis

Total RNA isolation was performed with the QuickRNA-Kit (Zymo Research Europe, Freiburg, Germany) according to the manufacturer’s instructions and quantified photometrically at 260 and 280 nm using a plate reader. An OD_260 nm/280 nm_ of 1.9–2.1 was considered protein-free RNA. To eliminate residual genomic DNA contamination, RNA samples were treated with DNase I according to the manufacturer’s protocol. cDNA synthesis was performed using the PrimeScript RT Master Mix (Takara Bio, Kusatsu, Japan) according to the manufacturer’s instructions. For cDNA synthesis, 1 μg RNA was added to 4 μL of 5X PrimeScript RT Master Mix, bringing the total volume to 20 μL with RNase-free ddH_2_O. The thermocycler was set for 15 min reverse transcription at 37 °C, followed by a 5 s inactivation step at 85 °C and a 4 °C hold. All primers used for subsequent quantitative PCR analyses were designed to span exon–exon junctions to prevent the amplification of genomic DNA.

### 4.6. Plasmid Preparation and Linearization

The following plasmids were used in this study: pRSFDUET-1-Hs-FNTalpha-Hs-FNTbeta1 (6240 bp), pRSFDUET-1-Hs-FNTalpha-GGT1B (6087 bp), pOTB7-RABGGTA (3748 bp), pDNR-LIB-RABGGTB (5687 bp), and pCMV-SPORT6-PTAR1 (5863 bp). Of these, the first two were constructed by cloning the coding sequences of *FNTA* and either *FNTB* or *PGGT1B* into a pRSF-DUET vector (kindly provided by Gerrit Praefcke); the other plasmids were obtained prefabricated from a commercial supplier (Horizon Discovery Biosciences Limited, Cambridge, UK). All plasmids were amplified in *Escherichia coli* DH5α and isolated using a standard plasmid miniprep protocol. Following isolation, plasmid DNA was linearized using the restriction enzyme NcoI according to the manufacturer’s instructions. Linearized DNA was purified using a commercial DNA clean-up kit (QIAquick PCR Purification Kit, Qiagen, Hilden, Germany) to remove residual enzyme and buffer components. Purified DNA was then quantified using a spectrophotometer and diluted to a working concentration of 1 ng/μL (for pOTB7-RABGGTA: 10 ng/μL) in nuclease-free water.

### 4.7. Reverse Transcriptase Quantitative PCR

Quantitative polymerase chain reaction (qPCR) amplification was performed using a CFX96 Touch Real-Time PCR Detection System (Bio-Rad, Hercules, CA, USA) and 96-well PCR plates. Each 20 µL reaction contained 10 µL of 2X iTaq Universal SYBR Green Supermix (Bio-Rad, Hercules, CA, USA), 1 µL of cDNA, 0.3 µL each of forward and reverse primers (10 pmol), and 8.4 µL of nuclease-free water (Fresenius Kabi, Bad Homburg, Germany). Plates were sealed and run on a C1000 Touch Thermal Cycler with the CFX96 Real-Time System. The amplification protocol consisted of an initial denaturation at 95 °C for 3 min, followed by 40 cycles of 95 °C for 10 s, annealing at the respective T_a_ for 30 s, and extension at 70 °C for 10 s. A melt curve analysis was conducted from 65 °C to 95 °C (0.5 °C increments, 5 s each) to verify amplicon specificity. All primers were designed to span exon–exon junctions and produce amplicons of 70–200 bp in length. Oligonucleotides were synthesized by Eurofins MWG Operon LLC.

Linearized plasmid DNA standards were used to generate a 6-fold serial dilution series to enable the absolute quantification of transcript levels. These dilutions were used to construct a standard curve for each gene of interest (GOI). After spectrophotometric determination of plasmid DNA concentration X, the copy number Y of standard DNA molecules was calculated using the following formula:

$$\frac{X\;g/µL\;\mathrm{DNA}}{MW\;(\mathrm{plasmid})}\;N_{A}=Y\;\mathrm{molecules}/µL$$

with MW being the molecular weight of the plasmid (as calculated with the NEBioCalculator, version 1.17.2, based on plasmid size or sequence; https://nebiocalculator.neb.com/#!/dsdnaamt, accessed on 18 July 2025), and *N*_A_ being the Avogadro constant of approximately 6.022 × 10^23^ mol^−1^. The standard curves for each gene were then constructed by plotting each dilution C_T_ value against the logarithm of its DNA amount, yielding a regression line with R^2^ > 0.95 for each gene (Supplementary Figure S1). The absolute transcript copy numbers of each sample were then determined by entering the samples’ C_T_ values into the equation of the regression line.

Amplification was performed for three biological replicates, each in technical duplicates across all experimental conditions.

### 4.8. Statistical Analysis

All statistical analyses were performed in Python (version 3.13) using the packages NumPy (version 2.3.0) and SciPy (version 1.16.0). Differences in DNA methylation between two groups (benign vs. malign) were assessed by unpaired *t*-test, and differences among all investigated cells were assessed using ANOVA followed by Dunnett’s test to compare methylation in each cancer cell line to that in whole blood, PBMCs, and HEK293 cells, yielding the mean difference between the compared samples and the 95% confidence interval (CI). The reported *p* values represent the multiplicity adjusted *p* values from Dunnett’s test. To evaluate the correlation between DNA methylation and gene expression, we determined the Pearson correlation coefficient (PCC). Nominal significance was assumed for *p* < 0.05.

## 5. Conclusions

The targeted analysis of DNA methylation in 19 cancer cell lines revealed no general differences between benign cells and cancer cell lines in DNA methylation regarding the promoter-associated CpG islands of the PTase genes *FNTA*, *FNTB*, *PGGT1B*, and *RABGGTA*. However, we observed significant differences between several individual cancer cell lines and certain control cells, most notably regarding the methylation of *FNTA*, *RABGGTA*, and *PGGT1B* in HEK293 cells and in the kidney cancer cell line Caki-1. The mean differences in methylation between individual cancer cell lines and benign controls ranged from +12.1 to −7.6 pp.

Among the benign and malign cells analyzed in this study, we observed a general inverse correlation of DNA methylation of *PGGT1B* and *PGGT1B* mRNA expression (PCC = −0.75; *p* = 0.005). Specifically, *PGGT1B* methylation in Caki-1 cells was lower than in HEK293, while its mRNA expression was higher. This observation confirms public data regarding mRNA expression in these cell lines and adds information about the possible causes for this differential expression. As *PGGT1B* expression is considered a validated prognostic factor in renal clear cell carcinoma, this discovery might become relevant for the management of renal clear cell cancer in the future.

Considering the significant yet relatively small differences in DNA methylation, general, biologically relevant modulation of PTase expression via DNA methylation in cancer cell lines seems unlikely. Nonetheless, we describe both distinct DNA methylation and DNA expression of *PGGT1B* in benign vs. malignant kidney cells, warranting a more focused investigation of the epigenetic regulation of *PGGT1B* in benign and malignant primary tissue from the kidney in a follow-up study.

To summarize, our results indicate the possibility of epigenetic regulation of *PGGT1B* expression via DNA methylation in a kidney cancer context and thus warrant further studies in primary tissues, especially regarding renal clear cell carcinoma.

## Figures and Tables

**Table 1:** Examples of aberrant PTase expression in cancer.

Genes	Observation	Potential Role in Cancer Etiology	Source
*FNTB*	overexpressed in ovarian cancer patients; overexpression correlates with K-Ras mutations	enhancement of defective Ras signaling	[4]
*FNTA* *FNTB*	overexpression in skin basal cell carcinoma patients	enhanced H-Ras processing	[6]
*FNTA* *FNTB*	tumor formation after overexpression in nude mice	enhanced Ras farnesylation	[9]
*PGGT1B*	overexpression in glioma patients	increased proliferation via enhanced Rac1 and RhoA prenylation	[7]
*RABGGTA* *RABGGTB*	overexpressed in colon, lung, and ovarian cancer as well as melanoma patients	regulation of endosomal trafficking via prenylation of Rab proteins	[5]

**Table 2:** DNA methylation of PTase genes and comparisons between benign and malign cells. For every gene, data are presented in percent as the mean methylation across all analyzed CpGs within the promoter-associated CpG island ± standard deviation. DNA methylation of all samples was compared using ANOVA, followed by Dunnett’s post hoc test comparing each cancer cell line to each benign sample (whole blood, PBMCs, and HEK293) as reference cells. For each significant difference, we show categorized *p* values ^a^, mean differences in methylation, and 95% confidence interval (in parentheses). Negative values indicate less methylation, whereas positive values indicate higher methylation relative to the reference cells. Differences reported as significant regarding all three reference cells are highlighted in bold. All numbers are given as percentage points.

Origin	Gene	*FNTB* (Mean of 8 CpGs)	Difference to Reference Cells			*FNTA* (Mean of 10 CpGs)	Difference to Reference Cells		
	**CpG location ^a^**	Chr14: 64,986,670 −64,986,749				Chr8: 43,056,611 −43,056,636			
	neg. control	4.3 ± 1.6%				2.2 ± 1.1%			
	pos. control	75.8 ± 15.0%				91.9 ± 7.7%			
			whole blood	PBMCs	HEK293		whole blood	PBMCs	HEK293
blood	whole blood	3.5 ± 0.9%	-		‡ −5.7 (−11.2 to −0.1)	4.3 ± 1.2%	-		‡‡ 2.4 (0.6 to 4.3)
blood	PBMCs	4.3 ± 0.8%		-		3.8 ± 0.8%		-	‡ 1.9 (0.1 to 3.8)
kidney	HEK293	9.2 ± 3.7%	* 5.7 (0.1 to 11.2)		-	1.9 ± 0.9%	** −2.4 (−4.3 to −0.6)	# −1.9 (−3.8 to −0.1)	-
kidney	Caki-1	6.8 ± 2.1%				**6.5 ± 2.7%**	* **2.2 (0.3 to 4.1)**	**##** **2.7 (0.8 to 4.6)**	**‡‡‡‡** **4.6 (2.7 to 6.5)**
kidney	A-498	6.2 ± 2.3%				4.1 ± 2.4%			‡ 2.2 (0.3 to 4.1)
bladder	T24	6.2 ± 1.9%				4.3 ± 1.7%			‡‡ 2.4 (0.5 to 4.3)
bladder	J82	5.8 ± 2.2%				3.4 ± 1.8%			
prostate	LNCap	7.8 ± 3.1%				4.6 ± 1.7%			‡‡ 2.7 (0.8 to 4.6)
prostate	PC3	5.0 ± 2.0%				2.3 ± 1.0%	* −2.0 (−3.9 to −0.2)		
prostate	DU 145	9.7 ± 5.1%	* 6.2 (0.6 to 11.7)			**6.5 ± 3.9%**	* **2.2 (0.3 to 4.0)**	**##** **2.7 (0.8 to 4.5)**	**‡‡‡‡** **4.6 (2.7 to 6.5)**
cervix	HeLa	8.1 ± 3.8%				2.4 ± 1.0%	* −1.9 (−3.8 to −0.0)		
ovaries	SK-OV-3	7.4 ± 2.0%				4.6 ± 2.2%			‡‡ 2.7 (0.8 to 4.6)
ovaries	NIH:OVCAR-3	5.2 ± 1.3%				4.7 ± 1.7%			‡‡‡ 2.8 (0.9 to 4.7)
ovaries	A2780	3.3 ± 0.9%			‡ −5.9 (−11.4 to −0.3)	5.1 ± 2.1%			‡‡‡‡ 3.2 (1.3 to 5.1)
chorion	JAR	7.1 ± 2.9%				4.2 ± 1.7%			‡‡ 2.3 (0.5 to 4.2)
liver	HepG2	10.0 ± 4.9%	* 6.5 (0.9 to 12.0)	# 5.7 (0.2 to 11.2)		6.0 ± 3.6%		# 2.2 (0.3 to 4.1)	‡‡‡‡ 4.1 (2.2 to 6.0)
blood	Jurkat	6.2 ± 1.0%				4.4 ± 1.9%			‡‡ 2.5 (0.7 to 4.4)
bone	U2OS	4.5 ± 1.2%				3.4 ± 1.4%			
neural crest	SK-N-AS	9.9 ± 5.2%	* 6.4 (0.8 to 11.9)	# 5.6 (0.1 to 11.2)		**6.3 ± 4.0%**	* **2.0 (0.1 to 3.9)**	**##** **2.5 (0.6 to 4.4)**	**‡‡‡‡** **4.4 (2.5 to 6.3)**
neural crest	SH-SY5Y	6.7 ± 2.1%				5.2 ± 2.4%			‡‡‡‡ 3.3 (1.4 to 5.2)
neural crest	Kelly	5.3 ± 1.6%				**9.0 ± 4.2%**	**** **4.7 (2.8 to 6.6)**	**####** **5.2 (3.3 to 7.1)**	**‡‡‡‡** **7.1 (5.3 to 9.0)**
neural crest	IMR-5	5.8 ± 2.3%				4.0 ± 2.5%			‡ 2.1 (0.3 to 4.0)
**Origin**	**Gene**	***RABGGTA*** **(Mean of 9 CpGs)**	**Difference to** **Reference Cells**			***PGGT1B*** **(Mean of 11 CpGs)**	**Difference to** **Reference Cells**		
	**CpG location ^b^**	Chr14: 24,271,320 −24,271,399				Chr5: 115,262,704 −115,262,816			
	neg. control	6.0 ± 2.4%				3.6 ± 1.1%			
	pos. control	78.8 ± 11.2%				72.7 ± 14.1%			
			whole blood	PBMCs	HEK293		whole blood	PBMCs	HEK293
blood	whole blood	5.4 ± 2.1%	-		‡‡‡‡ −6.0 (−8.0 to −4.0)	11.3 ± 6.9%	-	#### 5.9 (3.9 to 7.9)	
blood	PBMCs	3.9 ± 1.6%		-	‡‡‡‡ −7.5 (−9.5 to −5.5)	5.4 ± 1.1%	**** −5.9 (−7.9 to −3.9)	-	‡‡‡‡ −5.8 (−7.8 to −3.8)
kidney	HEK293	11.4 ± 4.1%	**** 6.0 (4.0 to 8.0)	#### 7.5 (5.5 to 9.5)	-	11.2 ± 2.2%		#### 5.8 (3.8 to 7.8)	-
kidney	Caki-1	5.0 ± 1.9%			‡‡‡‡ −6.4 (−8.4 to −4.4)	5.3 ± 1.2%	**** −6.0 (−8.0 to −4.0)		‡‡‡‡ −5.9 (−7.9 to −3.9)
kidney	A-498	**9.2 ± 3.4%**	**** **3.8 (1.7 to 5.7)**	**####** **5.3 (3.3 to 7.3)**	**‡** **−2.2 (−4.3 to −0.3)**	9.7 ± 2.2%		#### 4.3 (2.3 to 6.3)	
bladder	T24	5.0 ± 2.1%			‡‡‡‡ −6.4 (−8.4 to −4.4)	6.3 ± 1.4%	**** −5.0 (−7.0 to −3.0)		‡‡‡‡ −4.9 (−6.9 to −2.9)
bladder	J82	9.9 ± 3.3%	**** 4.5 (2.4 to 6.4)	#### 6.0 (4.0 to 8.0)		9.3 ± 2.0%		#### 3.9 (1.9 to 6.0)	
prostate	LNCap	9.5 ± 3.6%	**** 4.1 (2.0 to 6.0)	#### 5.6 (3.5 to 7.5)		**7.5 ± 1.9%**	**** **−3.8 (−5.8 to −1.7)**	**#** **2.1 (0.1 to 4.1)**	**‡‡‡‡** **−3.7 (−5.7 to −1.7)**
prostate	PC3	11.0 ± 7.1%	**** 5.6 (3.6 to 7.6)	#### 7.1 (5.1 to 9.1)		10.2 ± 7.1%		#### 4.8 (2.8 to 6.8)	
prostate	DU 145	13.3 ± 4.8%	**** 7.9 (5.9 to 9.9)	#### 9.4 (7.4 to 11.4)		11.4 ± 2.5%		#### 6.0 (4.0 to 8.1)	
cervix	HeLa	**16.0 ± 5.4%**	**** **10.6 (8.6 to 12.6)**	**####** **12.1 (10.1 to 14.1)**	**‡‡‡‡** **4.6 (2.6 to 6.6)**	11.7 ± 2.6%		#### 6.3 (4.3 to 8.3)	
ovaries	SK-OV-3	5.3 ± 2.0%			‡‡‡‡ −6.1 (−8.1 to −4.1)	6.8 ± 2.9%	**** −4.5 (−6.5 to −2.5)		‡‡‡‡ −4.4 (−6.4 to −2.4)
ovaries	NIH:OVCAR-3	9.7 ± 3.5%	**** 4.3 (2.2 to 6.2)	#### 5.8 (3.8 to 7.7)		**8.6 ± 2.1%**	** **−2.7 (−4.7 to −0.7)**	**###** **3.2 (1.2 to 5.2)**	**‡‡** **−2.6 (−4.6 to −0.6)**
ovaries	A2780	**8.4 ± 3.4%**	*** **3.0 (1.0 to 5.0)**	**####** **4.5 (2.5 to 6.5)**	**‡‡‡** **−3.0 (−5.0 to −1.0)**	6.0 ± 1.6%	**** −5.3 (−7.3 to −3.3)		‡‡‡‡ −5.2 (−7.2 to −3.2)
chorion	JAR	11.7 ± 4.5%	**** 6.3 (4.2 to 8.2)	#### 7.8 (5.8 to 9.8)		7.0 ± 1.7%	**** −4.3 (−6.3 to −2.3)		‡‡‡‡ −4.2 (−6.2 to −2.2)
liver	HepG2	**8.9 ± 3.7%**	**** **3.5 (1.4 to 5.4)**	**####** **5.0 (2.9 to 6.9)**	**‡‡** **−2.5 (−4.6 to −0.6)**	11.7 ± 2.5%		#### 6.3 (4.3 to 8.3)	
blood	Jurkat	4.5 ± 1.9%			‡‡‡‡ −6.9 (−8.9 to −4.9)	6.7 ± 1.5%	**** −4.6 (−6.5 to −2.5)		‡‡‡‡ −4.5 (−6.5 to −2.4)
bone	U2OS	5.1 ± 1.8%			‡‡‡‡ −6.3 (−8.4 to −4.4)	5.5 ± 1.3%	**** −5.8 (−7.8 to −3.8)		‡‡‡‡ −5.7 (−7.7 to −3.7)
neural crest	SK-N-AS	10.7 ± 4.4%	**** 5.3 (3.2 to 7.2)	#### 6.8 (4.8 to 8.8)		11.5 ± 2.5%		#### 6.1 (4.1 to 8.1)	
neural crest	SH-SY5Y	7.4 ± 2.9%		#### 3.5 (1.5 to 5.5)	‡‡‡‡ −4.0 (−6.0 to −2.0)	6.1 ± 1.3%	**** −5.2 (−7.2 to −3.2)		‡‡‡‡ −5.1 (−7.1 to −3.1)
neural crest	Kelly	3.8 ± 1.5%			‡‡‡‡ −7.6 (−9.6 to −5.6)	6.3 ± 1.2%	**** −5.0 (−7.0 to −3.0)		‡‡‡‡ −4.9 (−6.9 to −2.9)
neural crest	IMR-5	**9.1 ± 3.3%**	**** **3.7 (1.6 to 5.6)**	**####** **5.2 (3.2 to 7.2)**	**‡** **−2.3 (−4.4 to −0.4)**	**9.0 ± 1.8%**	* **−2.3 (−4.3 to −0.3)**	**####** **3.6 (1.6 to 5.6)**	**‡** **−2.2 (−4.2 to −0.2)**

^a^ Multiplicity-adjusted *p* values according to Dunnett’s post hoc test following ANOVA; *p* values of significant differences between a sample and different reference cells are indicated by different symbols for whole blood (*), PMBCs (#), and HEK293 (‡); */#/‡: *p* < 0.05; **/##/‡‡: *p* < 0.01; ***/###/‡‡‡: *p* < 0.001; ****/####/‡‡‡‡: *p* < 0.0001; ^b^: genome assembly GRCh38.p14.

## Data Availability

The datasets of the current study are available from the corresponding author upon reasonable request.

## Supplementary Materials

The following supporting information can be downloaded at: https://www.mdpi.com/article/10.3390/epigenomes10010017/s1, Figure S1: mRNA expression of PTase genes excluding whole blood samples as outliers.

## Abbreviations

The following abbreviations are used in this manuscript.

BSP	bisulfite-specific PCR
CI	confidence interval
DMEM	Dulbecco’s Modified Eagle Medium
EMEM	Eagle’s Minimum Essential Medium
FBS	fetal bovine serum
FPP	farnesyldiphosphate
FTase	farnesyltransferase
GGPP	geranylgeranyldiphosphate
GGPPS	GGPP synthase
GGTase I/II/III	geranylgeranyltransferase type I/II/III
GOI	gene of interest
NC	negative control
NTC	no-template control
PBMC	peripheral blood mononuclear cell
PC	positive control
PCC	Pearson’s correlation coefficient
PCR	polymerase chain reaction
PTase	prenyltransferase
qPCR	quantitative polymerase chain reaction
ROI	region of interest
RPMI	Roswell Park Memorial Institute
TSS	transcription start site
ULR	ultra-low range
