# Supplementary figures and images for: CpG Methylation of Protein Prenyltransferase Genes FNTA, FNTB, PGGT1B and RABGGTA in Cancer Cell Lines
Source: Epigenomes. 2026 Mar 4;10(1):17. doi: 10.3390/epigenomes10010017 (PMC13025263; doi:10.3390/epigenomes10010017)

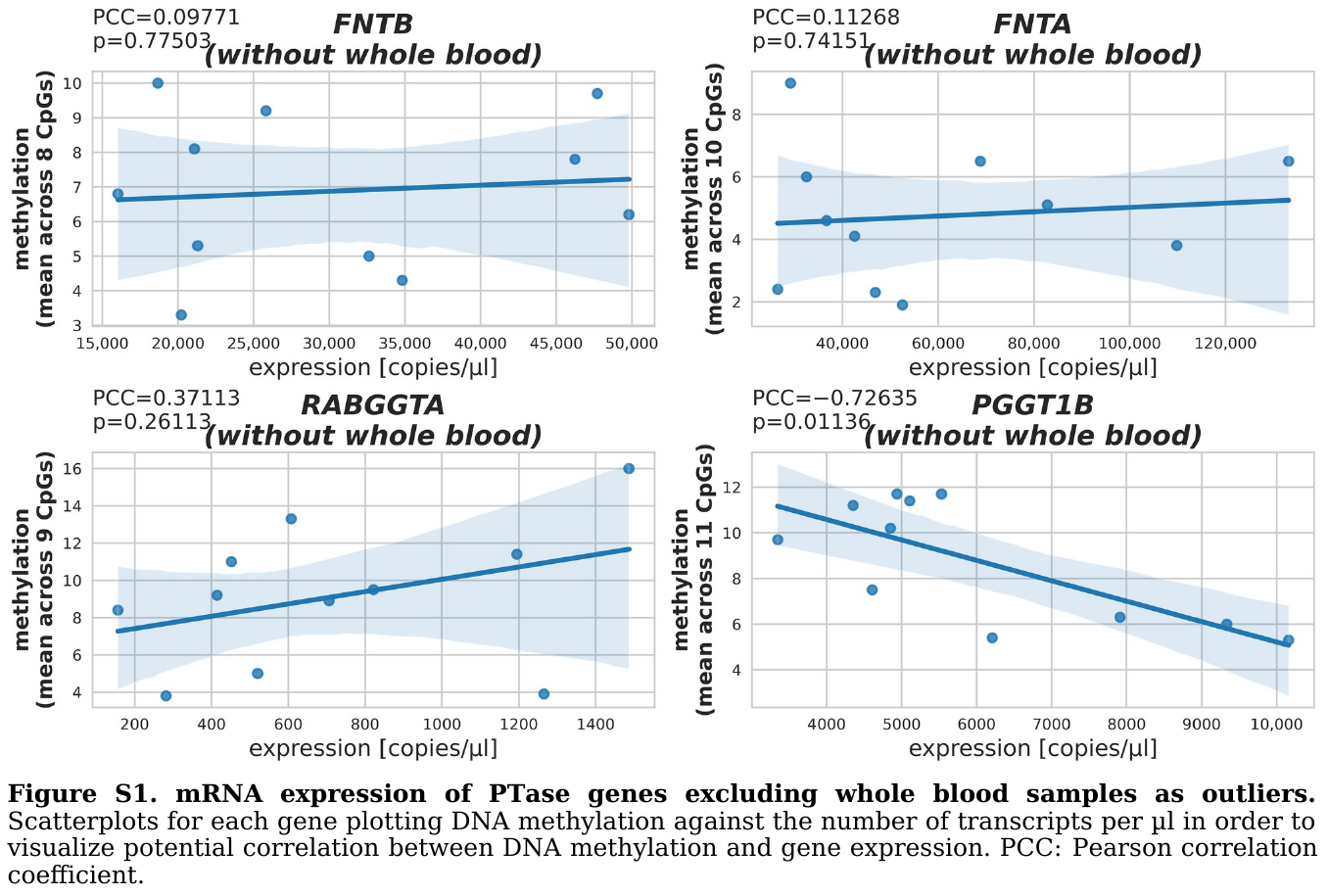

Supplement: Supplementary file 1 [file epigenomes-10-00017-s001.zip › epigenomes-4137560-supplementary.png]
